# Combinatorial code governing cellular responses to complex stimuli

**DOI:** 10.1038/ncomms7847

**Published:** 2015-04-21

**Authors:** Antonio Cappuccio, Raphaël Zollinger, Mirjam Schenk, Aleksandra Walczak, Nicolas Servant, Emmanuel Barillot, Philippe Hupé, Robert L. Modlin, Vassili Soumelis

**Affiliations:** 1 Department of Immunology, Institut Curie, Paris F-75248, France; 2 INSERM U932, Paris F-75248, France; 3 Centre for Cancer and Inflammation, Barts Cancer Institute, Queen Mary University of London, London EC1M 6BQ, UK; 4 Division of Dermatology, David Geffen School of Medicine at University of California, Los Angeles, California 90095, USA; 5 Laboratoire de Physique Théorique, CNRS, Université P. et M. Curie, École Normale Supérieure, Paris F-75248, France; 6 Institut Curie, Bioinformatique et Biologie des Systèmes, Paris F-75248, France; 7 INSERM U900, Paris F-75248, France; 8 Mines ParisTech, Fontainebleau, Paris F-77300, France; 9 CNRS UMR144, Paris F-75248, France; 10 Department of Microbiology, Immunology and Molecular Genetics, David Geffen School of Medicine at University of California, Los Angeles, California 90095, USA

## Abstract

Cells adapt to their environment through the integration of complex signals. Multiple signals can induce synergistic or antagonistic interactions, currently considered as homogenous behaviours. Here, we use a systematic theoretical approach to enumerate the possible interaction profiles for outputs measured in the conditions 0 (control), signals X, Y, X+Y. Combinatorial analysis reveals 82 possible interaction profiles, which we biologically and mathematically grouped into five positive and five negative interaction modes. To experimentally validate their use in living cells, we apply an original computational workflow to transcriptomics data of innate immune cells integrating physiopathological signal combinations. Up to 9 of the 10 defined modes coexisted in context-dependent proportions. Each interaction mode was preferentially used in specific biological pathways, suggesting a functional role in the adaptation to multiple signals. Our work defines an exhaustive map of interaction modes for cells integrating pairs of physiopathological and pharmacological stimuli.

## Abstract (web summary)

Cells constantly integrate information from multiple stimuli. By considering every possible means by which two stimuli can interact, Cappuccio *et al*. define 10 interaction modes and demonstrate their preferential use by dendritic cells responding to different combinations of microbial and host inflammatory cues.

## Graphical abstract

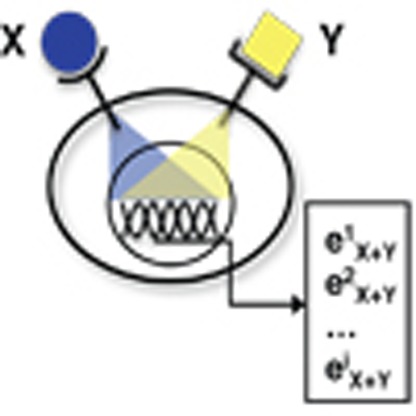

Signal integration is the process through which cells sense and compute the response to multiple information signals captured from their microenvironment. Since all cells evolve in complex and changing environments, this process is critical to their adaptation. For example, bacteria differentially sense various sources of carbon^1^, pH, antimicrobial peptides^2^,^3^ and nitric oxide^3^,^4^, each activating different pathways within their metabolic and genetic networks, which need to be integrated^5^. In higher order organisms, cells sense different signals, such as hormones, cytokines and physical properties of the extracellular matrix^6^,^7^,^8^. During inflammation, immune cells integrate a variety of microbial- and host-derived mediators^9^,^10^. In addition to the multiplicity of input signals, cells respond through multiple output adaptive responses, at the transcriptional, protein and phenotypic levels^3^,^11^,^12^, which further increases the complexity of the system.

A key aspect of the integration process is the possibility for interactions, when the effects of a combination of signals cannot be predicted from the effects of each agent acting alone. In this case, biologists and pharmacologists distinguish two scenarios: synergism and antagonism. In statistical terms, synergistic (or antagonistic) interactions occur if the fractional response to a combination of signals is significantly larger (or smaller) than the product of the fractional responses to each agent^13^. The classification of interactions as either synergistic or antagonistic has been extensively used to analyse the effect of combinations of biological stimuli and drugs^14^. This definition was also applied to multiple outputs assessed by medium- to high-throughput technologies^15^,^16^,^17^,^18^,^19^. Although synergistic and antagonistic behaviours are viewed as homogenous classes, combinatorial considerations related to the nonlinearity of signal interactions suggest that this conventional classification might confound a broad variety of biological scenarios.

Here we report an innovative system-level approach associating mathematical and data-driven analysis that allowed resolving the theoretical and experimental diversity of interactions, shedding new light on the integration process.

## Results

### Formalization of complexity underlying signal integration

**Figure 1:**
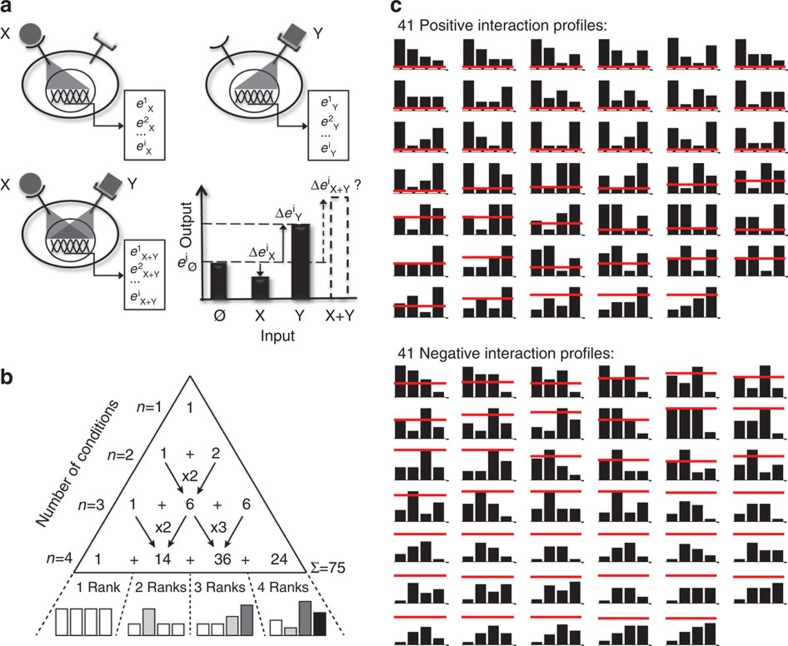
Theoretical analysis reveals 82 possible interaction profiles of two stimuli. (**a**) We consider two cues X or Y inducing the transcriptional states [*e*^1^_X_…e^i^_X_] or [*e*^1^_Y_…*e*^*i*^_Y_], where *e*^*i*^_X_ and *e*^*i*^_Y_ denote the expression of gene *i* due to X or Y. We hypothesize that after simultaneous triggering of the receptors for X and Y, the transcriptional state [*e*^1^_X+Y_…*e*^*i*^_X+Y_] cannot be predicted, as the downstream effects induced by both stimuli might interact either positively [Δ*e*^*i*^_X+y_>Δ*e*^*i*^_X_+Δ*e*^*i*^_Y_] or negatively [Δ*e*^*i*^_X+y_<Δ*e*^*i*^_X_+Δ*e*^*i*^_Y_]. (**b**) To enumerate the theoretical outcomes of an interaction, we first counted how many ways *k* statistically different groups can be observed from *n* experimental conditions. The generalized solution [*A*^*n*^_*k*_*=A*^*n−*1^_*k*_*+kA*^*n−*1^_*k−*1_] of this combinatorial problem is illustrated as a modified Pascal's triangle. Our experimental design involves *n*=4 conditions and therefore a maximum of *k*=4 statistically different groups, which results in 75 possible combinations. We then applied a constraint satisfaction algorithm to compute how many of these possibilities are mathematically consistent with the inequalities that define positive and negative interactions. We obtained 82 instances (41 positive and 41 negative) referred to as interaction profiles. (**c**) Tabular view of example profiles belonging to each of the 82 interaction profiles. The heights of the bars in each graph represent the expression levels of the four conditions (from left to right): No stimulus, X, Y and X+Y. The red line corresponds to the height of X+Y if the integration of the stimuli was additive.

First, we theoretically enumerated the possible interactions between two abstract signals X and Y. We denoted by **e**_**0**_, **e**_**X**_, **e**_**Y**_ and **e**_**X+Y**_ the log-normalized expression level of a multivariate output with *i* components measured, respectively, in the conditions 0 (control), signals X, Y and X+Y. Following log normalization, the fractional effects exerted by X, Y and X+Y on the *j*-th output component relative to the control condition correspond to the increments Δ*e*^*j*^_X_=*e*^*j*^_X_*−e*^*j*^_0_, Δ*e*^*j*^_Y_=*e*^*j*^_Y_*—e*^*j*^_0_, Δ*e*^*j*^_X+Y_=*e*^*j*^_X+*Y*_*—e*^*j*^_0_ (Fig. 1a), while the conditions of positive and negative interactions are translated, respectively, into the inequalities Δ*e*^*j*^_X+Y_>Δ*e*^*j*^_X_+Δ*e*^*j*^_Y_ and Δ*e*^*j*^_X+Y_<Δ*e*^*j*^_X_+Δ*e*^*j*^_Y_. For each output component independently, we used recursion analysis to enumerate the combinations of *k* statistically different groups (rankings) being observed from *n* experimental conditions. The generalized solution to this problem (*A*^*n*^_*k*_*=A*^*n−*1^_*k*_*+kA*^*n−*1^_*k−*1_) is illustrated as a modified Pascal's triangle (Fig. 1b). Our experimental design involves *n*=4 conditions (0, X, Y and X+Y), and thus a number of groups varying from a minimum of *k*=1 to a maximum of *k*=4. For example, for *n*=4, *k*=1 group there is only one possible profile where the output is constant in all conditions (Fig. 1b). By increasing to *k*=2, we add 14 non-redundant possible profiles corresponding to all combinations of the two output levels for the *n*=4 conditions. Summing each contribution up to a maximum of *k=*4, we obtained 75 possibilities (Fig. 1b). We then listed the profiles compatible with the inequalities Δ*e*^*j*^_X+Y_>Δ*e*^*j*^_X_+Δ*e*^*j*^_Y_ (positive interactions) and Δ*e*^*j*^_X+Y_<Δ*e*^*j*^_X_+Δ*e*^*j*^_Y_ (negative interactions). Five profiles were consistent with a lack of interaction (additivity) and were removed from subsequent analysis. Twenty-nine profiles were consistent with a positive, 29 with a negative and 12 were compatible with both a positive and a negative interaction. We then split each of these 12 ‘ambiguous' cases in two in order to account for both a positive and a negative realization. Altogether, we obtained 41 positive and 41 negative instances termed ‘interaction profiles' (Fig. 1c). We developed a constraint satisfaction algorithm in order to formalize the mathematical structure of the different profiles (Supplementary Fig. 1).

### Definition of 10 biological interaction modes

**Figure 2:**
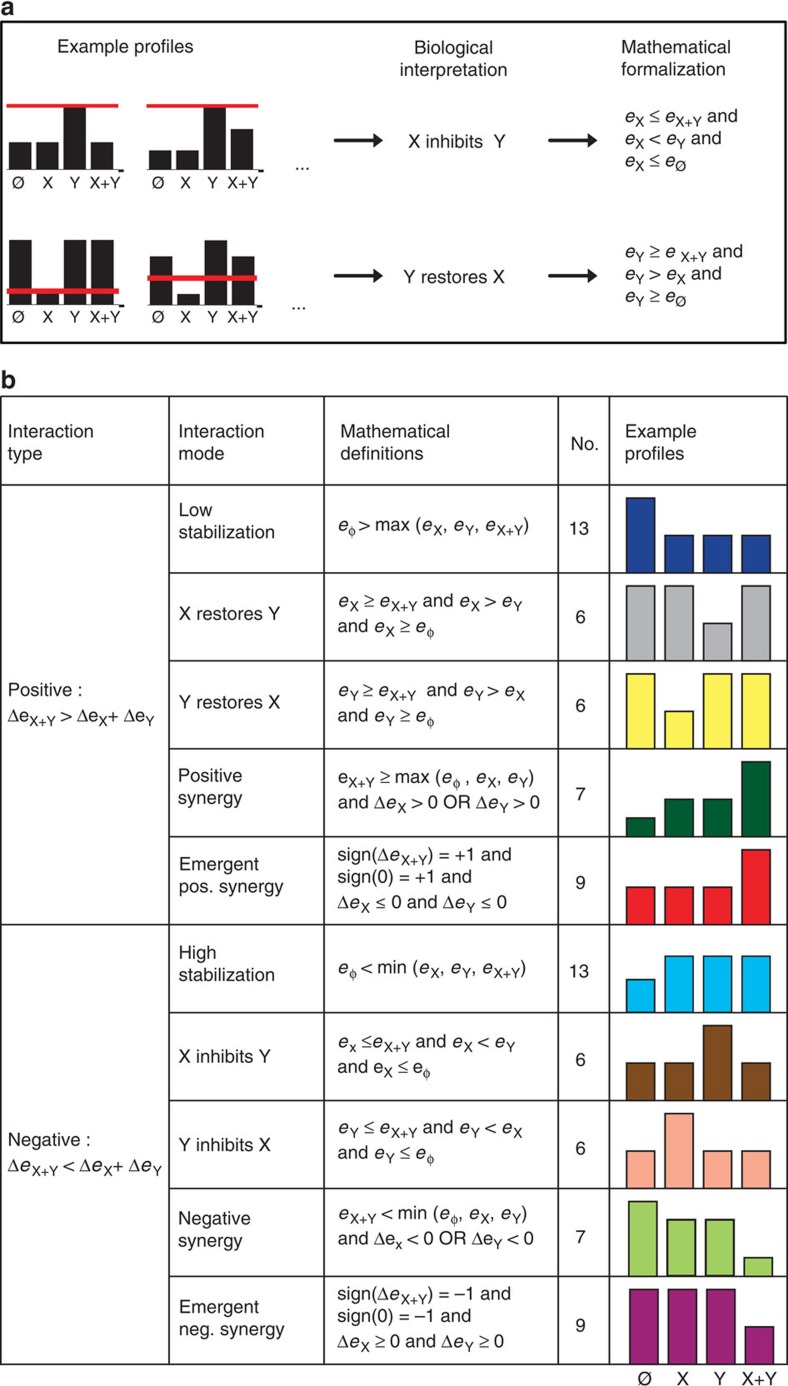
Classification of the 82 interaction profiles into 10 biological interaction modes. (**a**) Classification procedure on example interaction profiles belonging to the ‘X inhibits Y' and ‘Y restores X' interaction modes. The red line corresponds to the height of X+Y if the integration of the stimuli was additive. (**b**) Table showing the classification of the 82 interaction profiles in 10 groups called interaction modes. Each interaction mode contains a subset of interaction profiles that share a biological interpretation and satisfy common mathematical rules. The number of interaction profiles contained in each mode is shown as well as one representative interaction profile. Neg, negative; Pos, positive.

Our systematic combinatorial approach not only demonstrated that positive and negative interactions are highly heterogeneous classes but also showed that this broad diversity can be nailed down to a defined number of well-characterized behaviours. In an effort to simplify through identification of common patterns of behaviour, we observed that different interaction profiles could be interpreted as manifestations of similar biological effects, such as inhibition or restoration (Fig. 2a). Guided by biological interpretation and mathematical formalization, we reduced the 82 interaction profiles to 10 classes named interaction ‘modes' (Fig. 2b). The 41 positive interactions were classified in five modes as follows: ‘low stabilization', ‘X restores Y', ‘Y restores X', ‘positive synergy' and ‘emergent positive synergy' (Fig. 2b). The 41 negative interactions were classified in five additional modes as follows: ‘high stabilization', ‘X inhibits Y', ‘Y inhibits X', ‘negative synergy' and ‘emergent negative synergy' (Fig. 2b). In our classification scheme, each mode has a conjugated counterpart obtained by switching the sign of interaction, for example, ‘low stabilization' and ‘high stabilization'. Finally, we defined as opposite modes the pairs ‘X inhibits Y'/‘Y inhibits X' and ‘X restores Y'/‘Y restores X'.

### Multimodal signal integration in cultured dendritic cells

To explore the occurrence of interaction modes in a cellular system, we chose human innate immune cells because of the physiopathological importance of the integration process in these cells^10^. A first data set was generated using plasmacytoid pre-dendritic cells (pDCs), specialized in antiviral immunity^20^. PDC were stimulated with combinations of microbial- and host-derived signals, which coexist at sites of infection: interleukin (IL)-3 and influenza virus (Flu) (Toll-Like Receptor-7 ligand), GM-CSF (Granulocyte-Monocyte Colony Stimulating Factor) and Flu, and GM-CSF and antimicrobial peptide LL37 complexed with mammalian DNA (LL37/DNA, TLR-9 ligand)^21^. Transcriptional profiles were generated using Affymetrix microarrays in the conditions 0, X, Y and X+Y, at one or two time points for each pair of signals, in order to get a multiparametric assessment of output responses to combined stimuli. Quality control of the IL-3+Flu data set was performed using quantitative PCR (qPCR) on 20 genes, with median R2=0.98 between microarray and qPCR data (Supplementary Figs. 2a,b).

**Figure 3:**
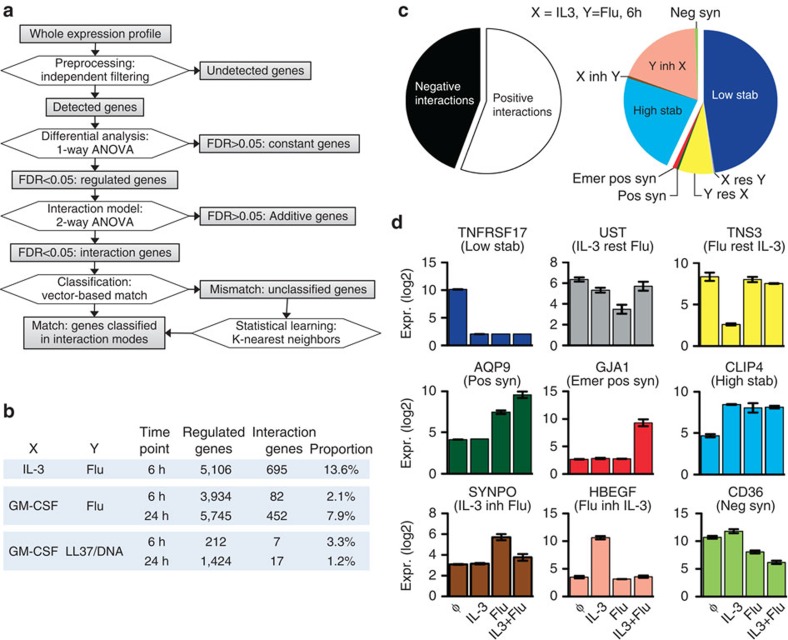
Multimodal signal integration in human pDC. (**a**) Gene classification flowchart showing the analysis steps from the whole transcriptome data to the classification of non-additive genes in the different interaction modes (IMs). (**b**) Number of regulated genes, interaction genes and the proportion of interaction genes with different stimuli combinations after 6 and 24 h of stimulation. (**c**) Distribution of IM for the combination IL-3 and Flu at 6 h, the number of genes per interaction mode was normalized to the number of interaction profiles per mode. Inh, inhibits; Res, restores. (**d**) Expression (Expr.) of example genes classified in different IM. Low stab, low stabilization; IL-3 rest Flu, IL-3 restores Flu; Flu rest IL-3, Flu restores IL-3; pos syn, positive synergy; emer pos syn, emergent positive synergy; high stab, high stablization; IL-3 inh Flu, IL-3 inhibits Flu; Flu inh IL-3, Flu inhibits IL-3; neg syn, negative synergy. Error bars represent s.e.m. of three 3 independent replicates.

In order to classify experimental gene expression profiles into one of the 82 theoretical profiles (and corresponding mode), we developed an original computational workflow consisting of the following steps (Fig. 3a): (1) data pre-processing, (2) selection of differentially expressed genes (one-way analysis of variance (ANOVA)), (3) identification of non-additive genes (two-way ANOVA model) and (4) classification of non-additive genes using vector-based approach or statistical learning. A false-discovery rate (FDR) of 0.05 was applied at both steps (2) and (3) for most subsequent analyses (see Methods section). The same framework can be used with a higher or lower FDR in order to further reduce false-negative or false-positive results, respectively.

Applying this workflow to the pDC data sets, we observed a range of 212–5,745 regulated genes, depending on the type of stimuli and on the time point (Fig. 3b). The number of non-additive genes ranged from 7 (3.3%) with GM-CSF and LL37/DNA at 6 h to 695 (13.6%) with IL-3 and Flu at 6 h (Fig. 3b). We focused on the IL-3 and Flu combination, showing the largest number of interactions. Using the final step of our flowchart (Fig. 3a), we mapped each non-additive response into one of the 10 interaction modes. As many as 9 out of the 10 modes occurred in pDC at the same time point (Fig. 3c), revealing a multimodal signal integration process, which meant that the same two signals were integrated according to different modes for different output genes. Decreasing the FDR to 1% lowered the total number of genes identified as interactions, as expected, but the multimodality was preserved, indicating a robust biological observation (Supplementary Fig. 3a,b). Importantly, both positive and negative interaction modes were observed for the same combination of signals, including conjugated modes such as ‘low stabilization' and ‘high stabilization' or ‘positive synergy' and ‘negative synergy' (Fig. 3c). However, except for a few cases, ‘Flu inhibits IL-3' excluded ‘IL-3 inhibits Flu' and ‘Flu restores IL-3' excluded ‘IL-3 restores Flu' (Fig. 3c), indicating that opposite modes tend to exclude each other. Although with comparatively low frequencies, we also detected positive and negative synergies, including emergent synergies. Of particular interest was the ‘Flu inhibits IL-3' class, which was unexpected given that both IL-3 and Flu are strong pDC activators, and there was no prior observation where one could inhibit the other. This indicated that a microbial signal may suppress effects of host-derived signals. Together with the classification of each gene into one of the interaction modes, we computed the Bliss factor^27^ that quantifies the strength of the interaction. The distribution of the Bliss factor for the four most represented modes within the Flu+IL-3 data set showed a marked heterogeneity (Supplementary Fig. 4). Although the Bliss factor is devoid of statistical value, it may be used to rank the significant interactions within a given mode in order to select extreme interaction behaviours. Interestingly, the ‘Flu inhibits IL-3' mode contained genes with remarkably large deviations from additivity (−6 to −7) in this data set (Supplementary Fig. 4).

Figure 3d shows genes representative of each mode identified in this data set. We could denote a close similarity between the theoretical (Fig.2b) and experimental (data-driven) interaction profiles, confirming the efficiency and accuracy of our classification workflow (Fig. 3a). Among genes of particular biological interest, *AQP9* (positive synergy) may facilitate pDC migration by promoting water flux^22^, while *GJA1* (emergent positive synergy) plays a role in cell–cell interaction and antigen transfer^23^. *HBEGF*, a cytoprotective agent strongly induced by IL-3, was not affected by Flu alone, but completely inhibited by the combination of IL-3 and Flu. CD36, a regulator of dendritic cell (DC) maturation and function^24^, displayed a peculiar antagonistic behaviour (Fig. 3d). Ingenuity pathway analysis (http://www.ingenuity.com/) showed that IL-3 and TLR transduction networks share the following five nodes: *ERK1* and *ERK2*, *c-Fos*, *c-Jun* and *Elk1*, which may suggest interactions (Supplementary Fig. 5). However, neither the frequency nor the diversity of such interactions could be anticipated.

Next, we asked whether multimodal signal integration by pDC was dependent on the nature of the stimuli. We analysed integrative data sets using a different pair of activating signals, GM-CSF and Flu (Supplementary Fig. 6). At 6 h, six interaction modes were observed as follows: ‘low stabilization', ‘Flu restores GM-CSF', ‘positive synergy', ‘emergent positive synergy', ‘high stabilization' and ‘Flu inhibits GM-CSF' (Supplementary Fig. 6a). Similar to IL-3 and Flu, conjugated modes were both represented, whereas opposite modes were mutually exclusive. At 24 h, diversity increased from 6 to 9 modes, with a more balanced representation of positive and negative interactions, and an increase in ‘high stabilization' (Supplementary Fig. 6b). This augmented diversity over time may be due to secondary autocrine and paracrine loops interfering with the exogenous stimuli.

Altogether, the pDC data sets revealed ‘low stabilization' and ‘high stabilization' as the most frequent modes. We performed additional analyses to address whether such behaviours may be due to technological aspects related to the methodology and mRNA read out used for our study. Results suggest that these modes cannot simply be explained by technological limitations due to detection limits or to saturation, respectively (Supplementary Fig. 7). For example, the level of high stabilization was in majority lower than the saturation limit of the microarray. We propose that these modes are driven in large parts by biological effects, such as stabilization of gene expression at specific levels and biological saturation of a given pathway or mRNA transcription, although they are also influenced by the technology used for data generation. In any case, it is crucial to identify and interpret these modes as distinct from other positive and negative interactions (such as synergies), because they represent very different outcomes.

### Multimodal integration of innate signals by human monocytes

**Figure 4:**
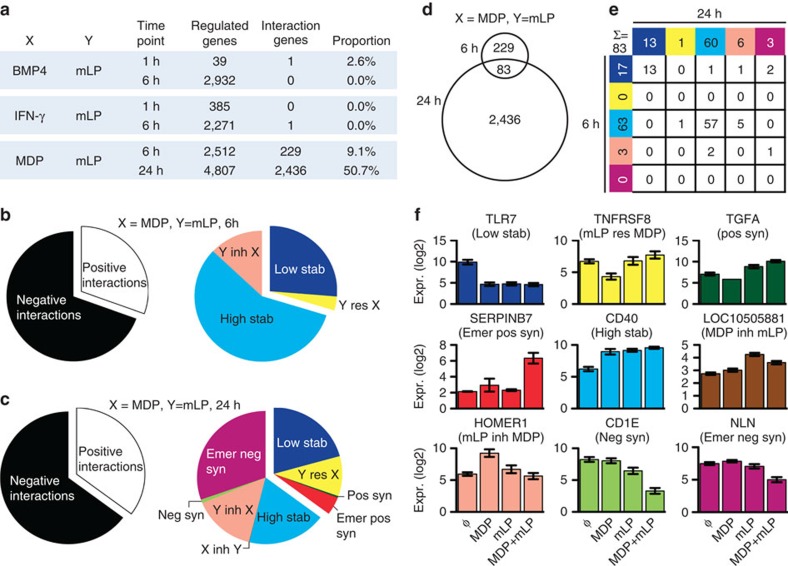
Multimodality and dynamics of the integration profile in human monocytes. (**a**) Number of regulated genes, interaction genes and the proportion of interaction genes with different stimuli combinations at 1, 6 and 24 h. BMP4, bone morphogenetic protein 4; IFN-γ, interferon-γ; MDP=muramyl dipeptide; mLP, 19 kDa mycobacterial lipopeptide. (**b**) Distribution of the integration modes proportions for the combination of MDP and mLP at 6 and 24 h (**c**), respectively. Inh, inhibits; Res, restores. (**d**) MDP and mLP interaction genes in common at 6 and 24 h. (**e**) Evolution of interaction genes classification over time. (**f**) Expression of example genes belonging to the different interaction modes. Low stab, low stabilization; mLP rest MDP, mLP restores MDP; pos syn, positive synergy; emer pos syn, emergent positive synergy; high stab, high stabilization; MDP inh mLP, MDP inhibits mLP; mLP inh MDP, mLP inhibits MDP; neg syn, negative synergy; Emer neg syn, emergent negative synergy. Error bars represent s.e.m. of five independent replicates.

To validate the occurrence of multimodal integration in another immune cell type, we analysed microarray data of human monocytes stimulated with combinations of microbial signals (MDP, muramyl dipeptide and mLP, mycobacterial lipopeptide), which activate the monocytes via NOD2 and TLR2/1, respectively, BMP4 (bone morphogenetic protein 4) and interferon (IFN)-γ (Fig. 4). These signals are relevant to the microenvironment of leprosy lesions and mycobacterial infections^25^,^26^. Two time points (6 and 24 h) were selected for each pair of ligands. The transcriptional profile of monocytes indicated efficient activation, as assessed by large numbers of regulated genes (Fig. 4a). Similar to pDC, we observed highly variable numbers and proportions of interactions, ranging from 0 (for BMP4+mLP and IFN-γ+mLP) to 2,436 (50.7%; for MDP+mLP; Fig. 4a). The combination of MDP+mLP resulted in a distribution of four interaction modes at 6 h (‘high stabilization', ‘low stabilization', ‘mLP inhibits MDP' and ‘mLP restores MDP'), which increased to nine modes at 24 h (Fig. 4b,c). By comparing the interaction genes at 6 and 24 h, we found 83 genes in common (Fig. 4d). These genes mostly remained within the same interaction mode (Fig. 4e), indicating an overall stability of the interaction modes over time. A shift from positive to negative interactions was observed in five genes (6%) and was mostly observed from low stabilization (positive interaction) to a negative interaction profile (Fig. 4e). The remaining 229 and 2,436 genes changed from interaction to additive and additive to interaction, respectively.

Contrary to pDC data sets, a majority of negative interactions were observed with a large proportion of emergent negative synergies at 24 h (Fig. 4c). However, the mutual exclusivity of opposite modes was even more apparent than in the pDC data sets. For example, ‘Y inhibits X' almost completely excluded ‘X inhibits Y' at both 6 and 24 h (Fig. 4c). Figure 4f shows representative genes for each interaction mode observed with the combination MDP+mLP at 24 h. *SERPINB7* (emergent positive synergy) not only may function as an inhibitor of Lys-specific proteases but also influences the maturation of megakaryocytes^27^. The costimulatory molecule *CD40* (high stabilization) was compatible with a plateau in DC maturation. The Major Histocompatibility Complex (MHC) -related *CD1E* (negative synergy) participates in the presentation of lipid antigens^26^.

In summary, we could establish multimodality in large-scale signal integration as a general principle occurring in different cell types responding to a diversity of combined stimuli.

### Coupling of interaction modes with cellular functions

**Figure 5:**
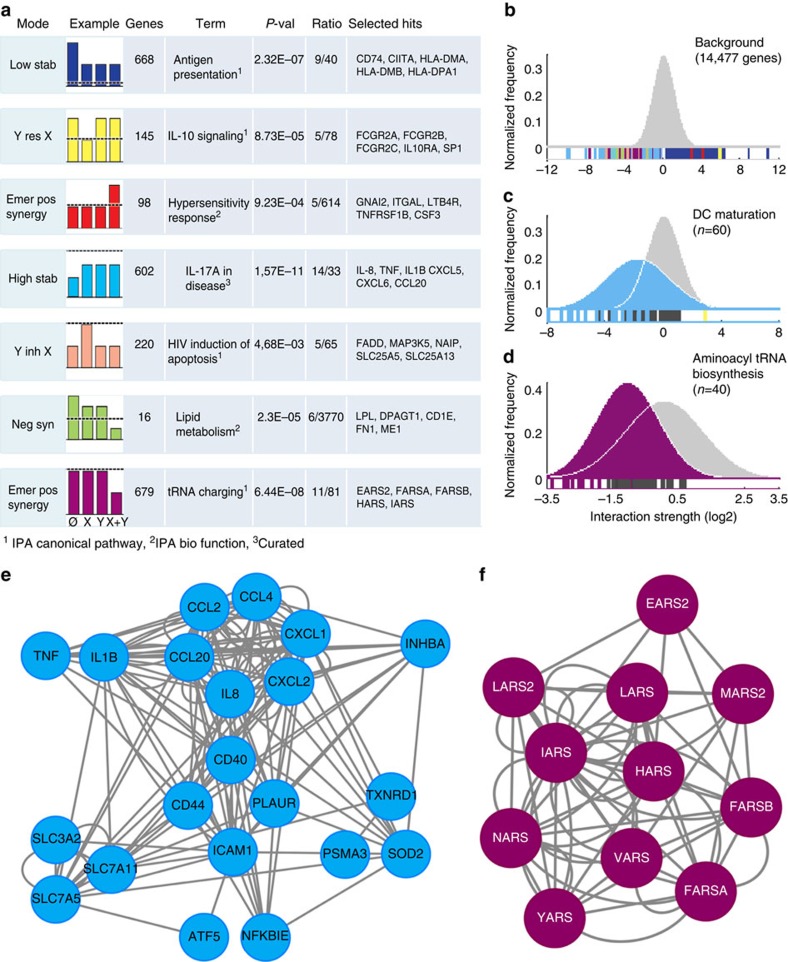
Selective integration mode imprinting of pathways and networks. (**a**) The table shows the top enriched canonical pathways or biological functions corresponding to each interaction mode. (**b**) The background distribution of the interaction strength (Δ*e*^*i*^_X+Y_−Δ*e*^*i*^_X_+Δ*e*^*i*^_Y_) for all expressed genes, modelled by a Gaussian distribution (continuous line). Each vertical bar corresponds to a gene whose colour code represents its classification in one interaction mode. Additive genes are in dark. (**c**) Distribution of the interaction strength calculated for the genes in the annotation terms ‘DC maturation' and (**d**) ‘tRNA aminoacyl biosynthesis', as compared with the background. The continuous lines represent a Gaussian fit to the empirical distributions, and the colour keeps track of the dominating mode in the corresponding annotation term. (**e**,**f**) Fully monomodal connected networks extracted from the classes ‘DC maturation' and ‘tRNA aminoacyl biosynthesis'. Abbreviations as in Fig. 4. IPA: Ingenuity Pathway Analysis.

To investigate its biological implications, we hypothesized that multimodal signal integration might enable cells to couple the integration process to specific functional modules. This would allow a refined adaptation to environmental changes tailored to each cellular function, and would imply that genes in each interaction mode would be enriched in specific biological functions. We tested this hypothesis on the monocyte MDP+mLP data set. Ingenuity pathway analysis revealed that seven modes were indeed enriched in genes contributing to specific functional pathways (Fig. 5a). For example, ‘high stabilization' was enriched in the ‘Th17 pathway in disease', not over-represented in other modes, while ‘emerging negative synergy' was enriched in the ‘tRNA charging pathway' (Fig. 5a). These findings supported the hypothesis that specific interaction modes underlie particular functional responses. This was validated using an additional of our own data sets on pDC integrating IL-3 and Flu (Supplementary Fig. 8), which revealed a significant enrichment in the pathway ‘NRF2-mediated oxidative stress' within genes belonging to the high stabilization mode and ‘coagulation system' within genes of the mode Y inhibits X. We also analysed a public microarray data set on monocytes integrating lipopolysaccharide (LPS) and anti-TREM1 (ref. 28) obtained from Gene Expression Omnibus (GEO; Supplementary Fig. 8). The pathways ‘*PPARa/RXRa* activation' and ‘role of hypercytokinemia' were significantly enriched in genes of the modes low- and high stabilization, respectively, but not in other modes. Overall, three independent data sets, obtained from various cell types and input signals, all point at a coupling between interaction modes and specific functional pathways.

To further explore the possibility of a link between interaction modes and specific functional responses, we developed an alternative strategy based on comparing the distribution of the Bliss Independence Index^29^ over a background of genes to its distribution over genes in given annotation terms. The distribution of this parameter for all genes could be approximated by a normal distribution (continuous curve in Fig. 5b), which served as reference background. We then generated analogous distributions for the genes contained in the MSigDB pathways (over 8,000 entries)^30^, and systematically compared them with the background using an information criterion (see Methods section). This allowed us to identify signalling and biological pathways showing consistently large interaction effects. Among those, we found terms almost exclusively dominated by specific interaction modes. For example, the terms ‘DC maturation' (Fig. 5c) and ‘aminoacyl tRNA' (Fig. 5d) were dominated by ‘high stabilization' and ‘emergent negative synergy', respectively. Interestingly, ‘DC maturation' was partially overlapping with the class ‘Th17 in disease', found independently with ingenuity pathway analysis (Fig. 5a), in particular for the genes *TNF*, *IL1B*, *IL8* and *CCL20*.

By mapping the hits in these terms onto a database of molecular interactions^31^, we could extract connected networks in both cases (Fig. 5e,f), which supports the existence of monomodal molecular networks. While the genes contained in these networks are a relatively minor fraction of the modes from which they are extracted (respectively, 21/602=3% and 11/679=2%), they cover a relevant fraction of the annotation term in which they are embedded (respectively, 21/60=35% and 11/40=27.5%).

The structure of the ‘DC maturation' network (Fig. 5e) revealed a coordinated regulation of multiple aspects related to the DC maturation process. Distinct subnetworks contained inflammatory cytokines (*IL1B* and *TNF*), chemokines (*IL8*, *CCL20*, *CCL2*, *CCL4*, *CXCL1* and *CXCL2*), surface molecules (*CD40*, *CD44*, *ICAM1* and *PLAUR*), amino acid transporters (*SLC3A2*, *SLC7A5* and *SLC7A11*) and transcription regulators (*ATF5* and *NFKBIE*; Fig. 5e). Thus, a single integration mode can be coupled to a functional pathway including a diversity of coordinated components.

## Discussion

Previous studies have used independence models to assess synergistic and antagonistic interactions in a quantitative manner based on phenotypic or molecular measurements^29^,^32^. For over 80 years, such models have remained a reference, although improvements have been made^13^. Our study revealed an unrecognized diversity in the possible interaction modes between two signals, within categories of behaviour previously thought to be homogeneous. Most importantly, it enabled to precisely quantify the spectrum of possible interactions and their link to particular biological functions.

To reach our objectives, we had to overcome the following four main challenges: (1) the enumeration of theoretically possible interaction profiles, (2) the classification of such profiles into biological interaction modes, (3) the projection of high-throughput data onto the theoretical space and (4) the link between interaction modes and biological functions.

A careful examination of the 82 elementary profiles obtained with combinatorial approaches led us to observe that common terms such as ‘inhibition', ‘synergy' and ‘restoration' could be realized in several ways. Guided by pattern-matching related to biological interpretation, and mathematical formalization, we could define 10 classes of biologically similar interactions termed interaction modes. While the definitions proposed in this work are not the only possible, they encompass all the theoretical space and they are objectively reproducible, which may help to promote a consensus terminology still lacking in the field of signal integration. Another advantage behind the introduction of biological modes is the possibility to gain biological insight, for example, by linking modes to specific functional pathways.

To explore the functional role of the different interaction modes, we performed enrichment analysis separately for each sufficiently represented mode. Our results, obtained from several independent data sets, suggest that each mode preferentially contributes to a particular function or pathway. However, we do not rule out the possibility for more complex scenarios. For example, different modes may participate in the same pathway, and/or the same mode may participate in different pathways.

To further elucidate the role of the interaction modes in shaping the global functional response of a cell to combined signals, it will be important to include the time dimension because, for example, the same genes may be classified in different modes at different times. Despite the fact that time series are not explicitly modelled in this work, our framework may still serve as a basic ‘grammar' for future extensions to the dynamic case. Other methodologies may be additionally used to account for the time factor, such as Bayesian methods^33^, Gaussian models^34^ or Fourier transformation^35^.

Although we used transcriptomics analysis to assess the multivariate cellular response, our methodological and conceptual frameworks do not rely on a specific type of measurement. Any quantitative output may be analysed for signal integration, at low- medium- or high throughput. As for each measurement method, transcriptomics data have an intrinsic noise^36^, which in our study did not prove major, since we obtained a very good correlation following qPCR validation (Supplementary Fig. 2). Importantly, for high-throughput data, our major conclusions were maintained using very stringent statistical criteria (1% FDR). Depending on the objective of each study, authors may decide to use lower or higher FDR according to whether they wish to privilege specificity or sensitivity, respectively^36^.

Current views generally consider that positive and negative interactions are mutually exclusive, that is, two signals cannot be synergistic and antagonistic^14^,^37^. Multimodality, as defined by the use of multiple modes in integrating the two same stimuli, implies a shift in our understanding, characterization and interpretation of signal integration from the nature of the signals to the output response that is considered. In other words, a simple assumption such as ‘signal X inhibits Y' does not stand unless explicitly associated to specific cellular outputs.

Previous large-scale studies have been instrumental in estimating the frequency of occurrence of an interaction between two signals for different cellular outputs^15^,^17^,^18^,^19^, which showed that high-throughput measurements are the most effective in capturing interactions. In our study, interactions were observed for 0–50% of output genes, and may have been missed or underestimated if only few output responses were measured. This, combined to the marked multimodality, further supports the use of large-scale, systems level assessment of output cellular responses in order to get a global and unbiased view of the integration process.

Our methodological framework may bring important biological insight through systematic evaluation of integration modes specific to a given cell type integrating physiopathologically relevant signals. We could uncover an unexpected inhibition of cytokine-induced pDC activation effects by TLR ligands, although both of these pDC stimuli exert strong activating effects when used as single agents. This ‘paradoxical inhibition' is in line with results on combinations of antibiotic drugs, showing that effective antibiotics may cross-inhibit each other, instead of increasing their potency^15^,^38^. Application of our framework to other cell types and pairs of signals should allow to address complex biological questions involving combinatorial stimulation, such as microbial hijacking of immune receptor cross-talk^39^, cytokine interactions in inflammatory diseases^40^ or immune checkpoint integration^41^.

The exhaustive map of interaction modes established in this study enables comparative studies of signal integration, in order to establish the relative contribution of the species, cell type, stimuli and cellular outputs, in determining the integration spectrum of multiple signals. It may also be applied to the integration of pharmacological agents in order to optimize drug combination strategies^14^,^42^ targeting specific cell types, such as in cancer or immune-mediated diseases.

## Methods

### Identification of the interaction profiles

The number of ways to rank the numbers *e*_0_, *e*_X_, *e*_Y_ and *e*_X+Y_ was computed using the recurrence relation *A*^*n*^_*k*_*=A*^*n−*1^_*k*_*+k A*^*n−*1^_*k−*1_ which, for *n=*4 and summing up for *k=*1, 2, 3 and 4 gives 75 possible rankings. Each ranking can be seen as a set of outcomes of the following qualitative pairwise comparisons:

1. *e*_X_ versus *e*_0_
2. *e*_Y_ versus *e*_0_
3. *e*_X+Y_ versus *e*_0_
4. *e*_Y_ versus *e*_X_
5. *e*_X+Y_ versus *e*_X_
6. *e*_X+Y_ versus *e*_Y_

In our framework, the outcome of a qualitative comparison can take on the following three values: 0 (equal numbers), 1 (first larger than the second) and −1 (first smaller than the second). Each of 75 rankings was coded uniquely as a vector of six components describing the qualitative outcome of the comparisons 1–6 as listed above. For example, the vector (0,0,1,0,1,1) corresponds to:

*e*_X_=*e*_0_, *e*_Y_=*e*, *e*_X+Y_>*e*_0_, *e*_Y_=*e*_X_, *e*_X+Y_>*e*_X_, *e*_X+Y_> *e*_Y_

To identify which rankings were compatible with positive or negative interactions, we considered the equations 1–6 together with the inequalities that define positive and negative interactions:

1. Δ*e*_X+Y_ *>*Δ*e*_X_ *+*Δ*e*_Y_ (positive interaction)
2. Δ*e*_X+Y_ *<*Δ*e*_X_ *+*Δ*e*_Y_ (negative interaction)

which can be written as

(7a) *e*_X+Y_ −*e*_X_−*e*_Y_+*e*_0_>0

(8a) *e*_X+Y_ −*e*_X_−*e*_Y_+*e*_0_<0

If a ranking is consistent with a positive (or negative) interaction, the inequality constraints encoded in the corresponding vector can be solved simultaneously with 7a (or 8a). To verify this, we developed a constraint satisfaction that attempts to solve the six constraints of each ranking together with the inequality 7a (or 7b). The solution is searched numerically with a MATLAB linear solver. The variables *e*_0_, *e*_X_, *e*_Y_ and *e*_X+Y_ were constrained to the interval 2–16, taken as an approximate of the range of expression values from Affymetrix chips in log2 scale. The method is implemented in the MATLAB code and is available upon request.

### Classification algorithm

To classify the measured expression levels of the genes into the interaction modes, we developed a MATLAB algorithm consisting of the following steps:

1. For all genes: identification of differentially expressed genes. This is done with a one-way ANOVA (MATLAB function *anova1*), and subsequent correction of the *P* values through the function *mafdr* (Benjamini–Hocheberg) and a significance threshold of 5%.
2. For differentially expressed genes: identification of non-additive genes. This step is done with a two-way ANOVA (MATLAB function *anova2*), and subsequent correction of the *P* values through the function *mafdr* (Benjamini–Hocheberg) and a significance threshold of 5%.
3. For non-additive genes: match between theoretical and experimental interaction profiles.

To obtain the match, we performed the pairwise comparisons in 1–6 in statistical terms by calculating the confidence interval (CI) of the differences of all the group means. Consistent with the theoretical analysis, a comparison between two experimental groups was assigned 0 if the CI contained 0; 1, if the CI was always positive; and −1, if the CI was always negative. This allowed producing a six-dimensional vector for each gene, which we matched with one of the vectors defined theoretically. If, due to statistical inconsistencies, a non-additive gene could not be classified in any of the theoretical profiles, it was assigned to the class containing its nearest neighbour. Such step was done using a *k*-nearest neighbour algorithm (MATLAB function *knnclassify*) with the correlation distance. We refer to these cases as ‘learned interaction profiles'.

The above workflow was implemented in a MATLAB function available upon request.

### Functional interpretation of the interaction modes

Enrichment analysis of the gene lists corresponding to the interaction modes was done with ingenuity pathway analysis. For pathway differential analysis, for each gene *i*, we calculated the parameter *s*^*i*^*=<Δe*^*i*^_X+Y_*>*−*<Δe*^*i*^_X_*>+<Δe*^*i*^_Y_*>*, where the symbol <·> denotes the average with respect to multiple donors and *i*=1, …, *n*. The number *n* is the size of the background, which was chosen as the set of all genes passing the step of independent filtering. The parameter *s*^*i*^ measures the deviation from additive integration and can thus be defined as the average interaction strength for gene *i*. The background distribution of *s*^*i*^ can be modelled by a Gaussian function with average *μ*_b_, and s.d. *σ*_b_, where the subscript refers to background. The parameters of the background distribution were estimated by maximizing the maximum Likelihood criterion.

Next, we downloaded the Molecular Signature Database (MSigDB, Broad Institute). For each term having at least 10 members, we computed the distribution of the interactions strength. The within-term distributions again modelled as normal functions with parameters *μ*_p_ and *σ*_p_, and compared with background using the Akaike Information Criterion (AIC) corresponding to the following alternatives:

1. The background distribution and the within-term distribution can be described by the same normal function (*μ*_p_ *=μ*_b_ *=μ*, *σ*_p_ *=σ*_b_ *=σ*). In this case, we have AIC*=*(*n+m*)(log(2*πσ* ^2^)*+*1)*+*2 × 2,
2. The background distribution and the within-term distribution can be described by two normal functions with different average but same s.d. (*μ*_p_ *≠ μ*_b_, *σ*_p_ *=σ*_b_ *=σ*)AIC*=*(*n+m*)(log(2*πσ* ^2^)*+*1)*+*2 × 3
3. The background distribution and the within-term distribution can be described by two normal functions with same average but different s.d. (*μ*_p_ *=μ*_b_ *=μ*, *σ*_p_ *≠σ*_b_)AIC*=*(*n+m*)(log(2*π*)*+*1)*+n*log(*σ* ^2^_b_)*+m*log(*σ* ^2^_p_) 2 × 3
4. The background distribution and the within-term distribution can be described by two normal functions with different average and different s.d.'s. There are four parameters (*μ*_p_ *≠μ*_b_, *σ*_p_ *≠σ*_b_)AIC*=*(*n+m*)(log(2*π*)*+*1)*+n*log(*σ* ^2^_b_)*+m*log(*σ* ^2^_p_) 2 × 4.

The optimal scenario was identified as resulting in the minimum AIC.

### PDC purification and culture

Blood buffy coats from healthy human donors were obtained from Etablissement Français du Sang, Paris, Saint-Antoine Crozatier blood bank through an approved convention with the Institut Curie. All donors gave their informed consent for research use of the blood samples. Experimental procedures with human blood have been approved by the Curie Hospital Ethical Committee for human research and were performed in accordance with European Union guidelines and the Declaration of Helsinki. Peripheral blood mononuclear cells (PBMCs) were isolated using Ficoll gradient (Amersham). Fresh pDCs were isolated from PBMCs using the negative selection pDC untouched isolation kit (Miltenyi Biotec) followed by fluorescence-activated cell sorting using staining (CD11c−, CD4+ and CD123+; all obtained from BD Biosciences). pDC purity was over 98%. Freshly isolated pDC were cultured in RPMI 1640 (Invitrogen) containing 10% fetal bovine serum (Hyclone) for 6 h at 37 °C and 5% CO_2_ in culture medium alone or supplemented with the following compounds or combinations: Flu, which is heat-inactivated influenza virus at a concentration of 10E7 PFU per ml (PR8 strain; Charles River Laboratories). IL-3 and GM-CSF were both used at a concentration of 10 ng ml^−1^ (R&D Systems). And LL37 coupled to DNA (from PBMC, *Escherichia coli* digested). Synthetic LL37 (Innovagen) was premixed with human genomic DNA as described by Lande *et al*.^43^ Final concentration used for LL37 (50 μg ml^−1^) and for the DNA (10 μg ml^−1^).

### PDC RNA extraction and evaluation

Total RNA was extracted with RNeasy Micro kit (Qiagen) including on-column DNase digestion. RNA concentration and absence of protein contamination were determined using the NanoDrop instrument. All RNA samples had 260 nm/280 nm absorbance ratios between 1.9 and 2.1, indicating high purity. RNA quality was assessed using RNA 6000 Nano chips on the Agilent 2100 Bioanalyzer. Only samples with RIN>7 were further used for gene chip hybridization and qPCR.

### PDC gene chip data generation

RNA amplification and labelling was performed according to the protocol recommended by Affymetrix with 100 ng initial totRNA per sample. Samples were hybridized to Affymetrix Human Genome U133 Plus 2.0 arrays. This pDC Affymetrix data are already submitted to GEO and access can be given to the reviewers. Sample preparation, hybridization, washing, staining and scanning was performed by the Institut Curie Gene Chip core facility. The raw data.CEL files were imported into Partek Genomics Suite software for normalization with the GC-RMA algorithm. For all subsequent analyses, the log2 of expression values were used. Then, independent filtering of the data was performed with each data set independently and consisted of following steps: (1) for genes with multiple probe sets, only the probe set with the highest average value was retained. (2) Genes not detected in any experimental condition were removed from the data set. In this study, we used exclusively data generated with the HG U133 Plus 2.0 arrays, for which we defined the detection threshold to be 4. Every replicate of at least one condition had to be >4, for a given gene to be selected for further analysis. This pre-processed data was then imported into MATLAB for further computational analysis.

### Quantitative reverse transcription PCR

cDNA was synthesized with a mix containing random hexamers, oligo(dT)15 (Promega) and SuperScript II reverse transcriptase (Invitrogen). Transcripts were quantified by real-time quantitative reverse transcription PCR on Light Cycler 480 (Roche) with Applied Biosystems predesigned TaqMan Gene Expression Assays and Absolute qPCR ROXmix (Thermo Fisher Scientific). All cycle thresholds (Cts) were normalized to the housekeeping gene *B2M* (*beta 2 microglobulin*) by subtracting the target gene Ct from the housekeeping gene Ct. In that way, the qPCR data are represented as a raw delta Ct value, which is a log2 relative expression scale. This simple way to calculate relative expression values enables direct comparison of expression differences between two conditions in qPCR and the log2 Affymetrix values. The following gene expression assays were used: *ATP5O*: Hs00426889_m1; *CCL3*: Hs00234142_m1; *CCL4*: Hs99999148_m1; *CCL5*: Hs00982282_m1; *CCND2*: Hs00277041_m1; *CD86*: Hs00199349_m1; *CXCL10*: Hs00171042_m1; *CXCL11*: Hs00171138_m1; *DUSP6*: Hs01044001_m1; *EEF1A1*: Hs00742749_s1; *FOXP1*: Hs00212860_m1; *GJA1*: Hs00748445_m1; *ICOSLG*: Hs00323621_m1; *IFNA2*: Hs00265051_s1; *IL1B*: Hs00174097_m1; *IL6*: Hs00174131_m1; *IL8*: Hs00174103_m1; *NDUFA1*: Hs00244980_m1; *TNF*: Hs00174128_m1; *TNFRSF17*: Hs00171292_m1 and *B2M*: Hs99999907_m1.

### Monocytes purification and culture

This data was published by Schenk and colleagues^25^ and is accessible through the GEO number: GSE34156.

Briefly, whole blood from healthy donors (UCLA I.R.B. no. 92-10-591-31) with informed consent was obtained. PBMCs were isolated using Ficoll (GE Healthcare) gradient centrifugation and monocytes were further purified using negative selection with microbead-coupled IgG (Miltenyi Biotec). Cells were cultured for in RPMI and 10% fetal calf serum (FCS) (Omega Scientific). For activation via TLR2/1, the 19-kDa mLP (EMC Microcollections) was used at a final concentration of 1 μg ml^−1^ in all experiments. BMP4 was used at 100 ng ml^−1^ (R&D Systems).

## Author contributions

A.C. developed the mathematical, statistical and computational analyses of the work, contributed to figures design and to the writing of the manuscript. R.Z. designed and carried out pDC experiments, contributed to computational analyses, created figures and contributed to manuscript writing. M.S. carried out the ingenuity computational analyses and monocyte experiments. A.W. contributed to the mathematical analysis necessary to enumerate the interaction profiles. N.S., E.B., P.H. and R.L.M. contributed to study design and computational analyses. V.S. supervised the study, contributed to the figures design and computational analyses, and wrote the manuscript. All co-authors participated in discussions over the results and commented on the original manuscript.

## Additional information

**How to cite this article:** Cappuccio, A. *et al*. Combinatorial code governing cellular responses to complex stimuli. *Nat. Commun.* 6:6847 doi: 10.1038/ncomms7847 (2015).

## Supplementary Material

Supplementary Information Supplementary Figures 1-8
