# Supplementary material for: Combinatorial code governing cellular responses to complex stimuli
Source: Nat Commun. 2015 Apr 21;6:6847. doi: 10.1038/ncomms7847 (PMC4410637; doi:10.1038/ncomms7847)
Supplement: Supplementary Information — Supplementary Figures 1-8 [file ncomms7847-s1.pdf]

a

b

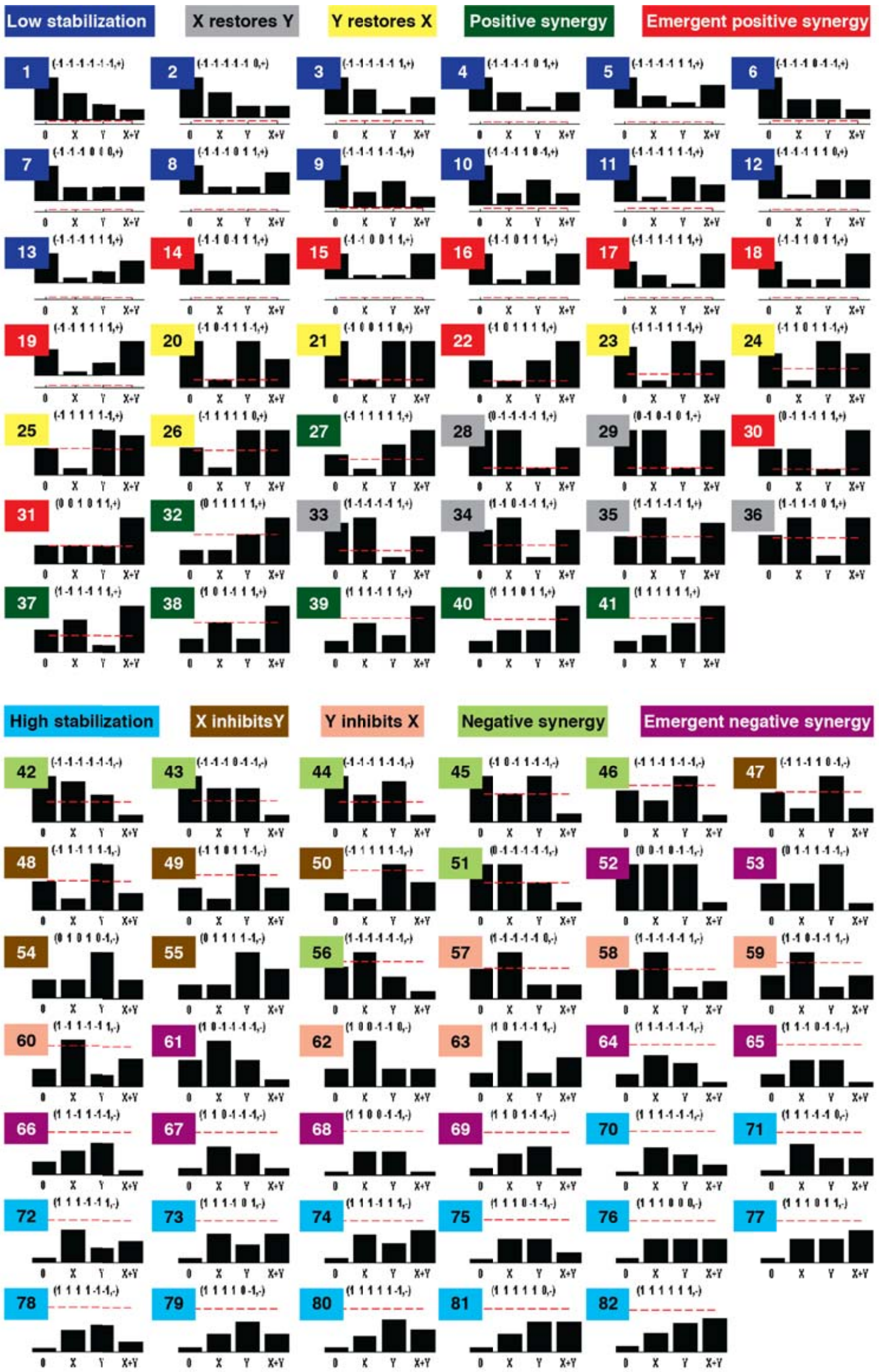

Supplementary Figure 1: The qualitative behaviour of the 82 Interaction profiles and their classification in the interaction modes. Related to Figure 1. a, The vectors composed of 6 numbers (-1, 0 or 1) and a sign (+ or -) uniquely define each of the interaction profiles correspond to the outcome of the following comparisons: X vs 0, Y vs 0, X+Y vs 0, Y vs X, X+Y vs X and X+Y vs Y. The dashed red line corresponds to the expression level of e_{X+Y} if the profile was additive. Graphs 1 to 41 represent positive interaction profiles. b, Graphs 42-82 represent negative interaction profiles.

a

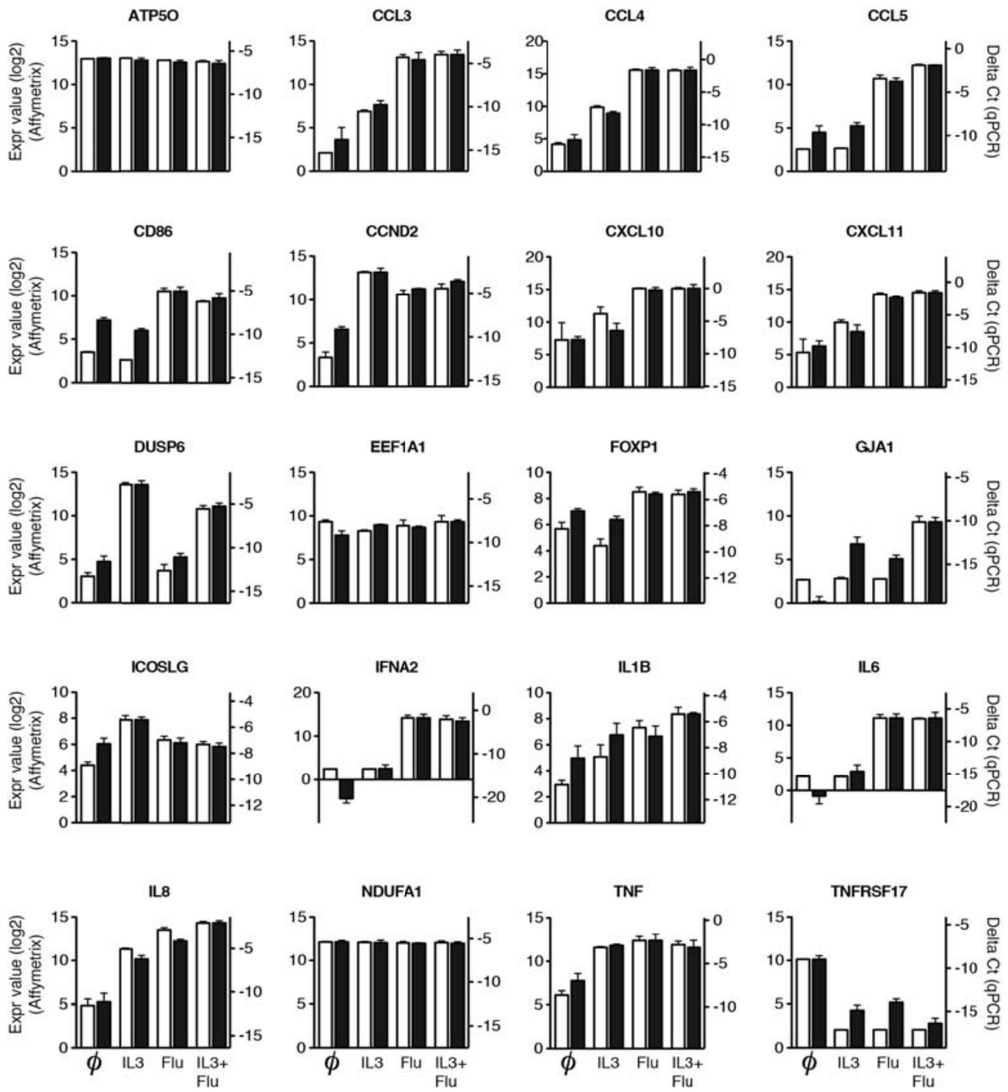

b

Gene	R2	Gene	R2
ATP5O	0.706	FOXP1	0.988
	0.999	A1	489
CCL4	0.986	ICOSLG	0.633
	0.988	A2	902
CCND2	0.997	IL1B	0.821
	0.980		934
CXCL10	0.904	IL8	0.968
	0.947	UFA1	944
DUSP6	1.000	TNF	0.989
	0.082	FRSF17	907
		**MEDIAN R2**	**0.983**

Supplementary Figure 2: Validation of Affymetrix gene chip data by quantitative RT PCR (qPCR). a, A sample of 20 genes representing different types of profiles and expression intensities was chosen to be confirmed in the experimental setting pDC, X=IL3, Y=Flu, 6h. The empty bars represent the average Affymetrix expression values (refer to left Y axis). The error bar is the standard error (SE) of 3 biological replicates. The black bars represent the qPCR data obtained with the same experimental setting but with pDC from 3 different donors than the ones used for the gene chip hybridization. The height of the black bars represent the average of the delta Cts which are obtained by subtracting the Ct of the target gene from the Ct of the normalizing gene B2M. The right Y axis indicates the delta Ct levels. This axis was shifted so the level of the highest qPCR value matches the level of highest gene chip value to facilitate the comparison of the profiles. The height and range of the left and right Y axis are the same. **b**, Squared correlation coefficients between Affymetrix and qPCR profiles are shown for each of the 20 genes.

a

X	Y	Time point	Regulated genes	Interaction genes	Proportion
IL3	Flu	6h	3115	214	6.8%
GMCSF	Flu	6h	2074	29	1.4%
		24h	3635	105	2.9%
GMCSF	LL37/DNA	6h	26	2	7.7%
		24h	67	7	10.4%

b

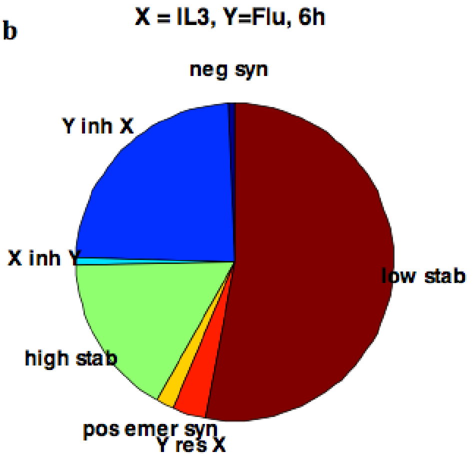

c

X	Y	Time point	Regulated genes	Interaction genes	Proportion
BMP4	mLP	1h	1	0	0%
		6h	1128	0	0%
IFN-γ	mLP	1h	142	0	0%
		6h	461	0	0%
MDP	mLP	6h	1535	119	7.7%
		24h	2725	1024	37.5%

d

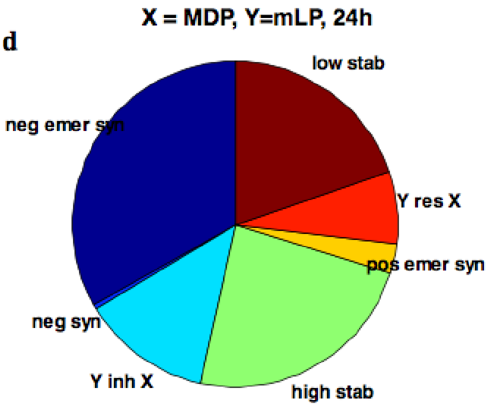

Supplementary Figure 3: Multimodal Signal integration with an FDR=1% for both ANOVA1 and ANOVA2 tests. a, Number of regulated genes, interaction genes, and the proportion of interaction genes with different stimuli combinations after 6 and 24 hours of stimulation in pDC and **c**, monocytes. **b**, Distribution of Interaction Modes for the combination IL3 and Flu at 6h, and **d**, monocytes at 24h.

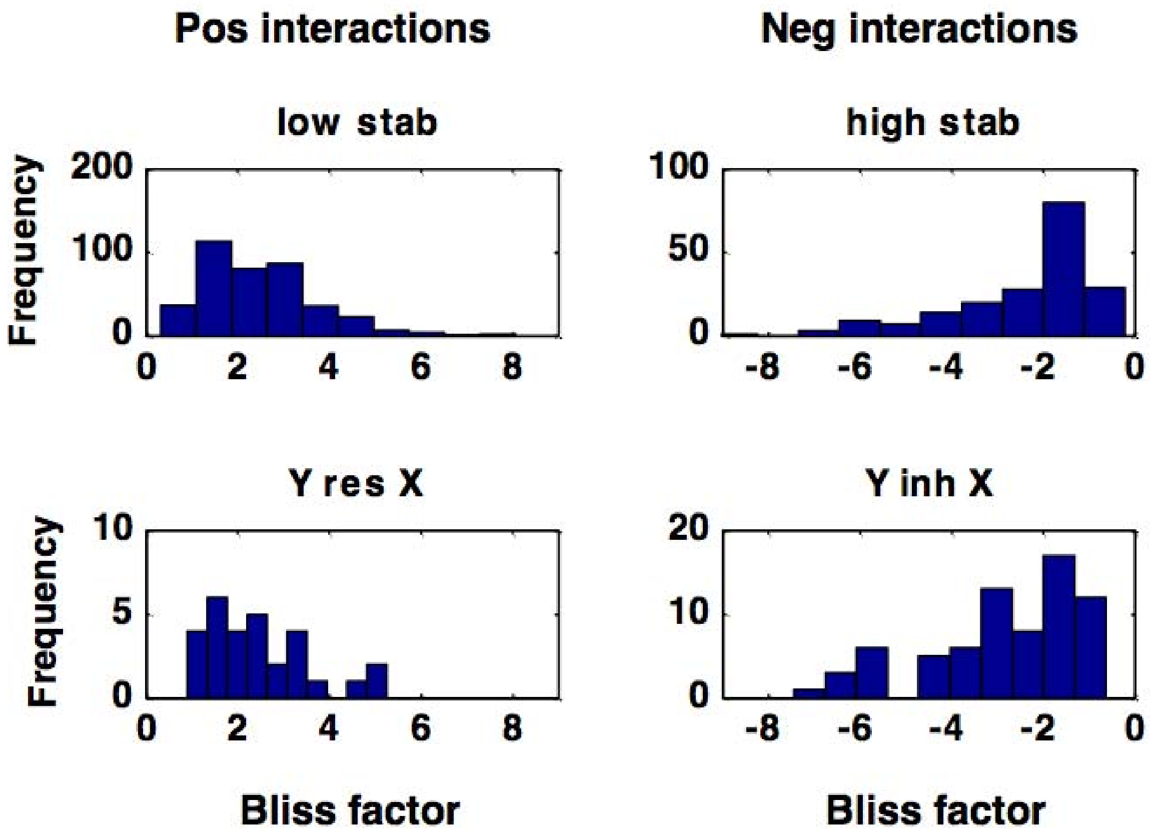

Supplementary Figure 4: Bliss Factor distribution in the pDC data set. Histograms show frequency distributions of the Bliss factor, which measures the deviation from additive integration, calculated separately for the four most represented interaction modes found in the dataset of pDC integrating IL3 and Flu at 6h.

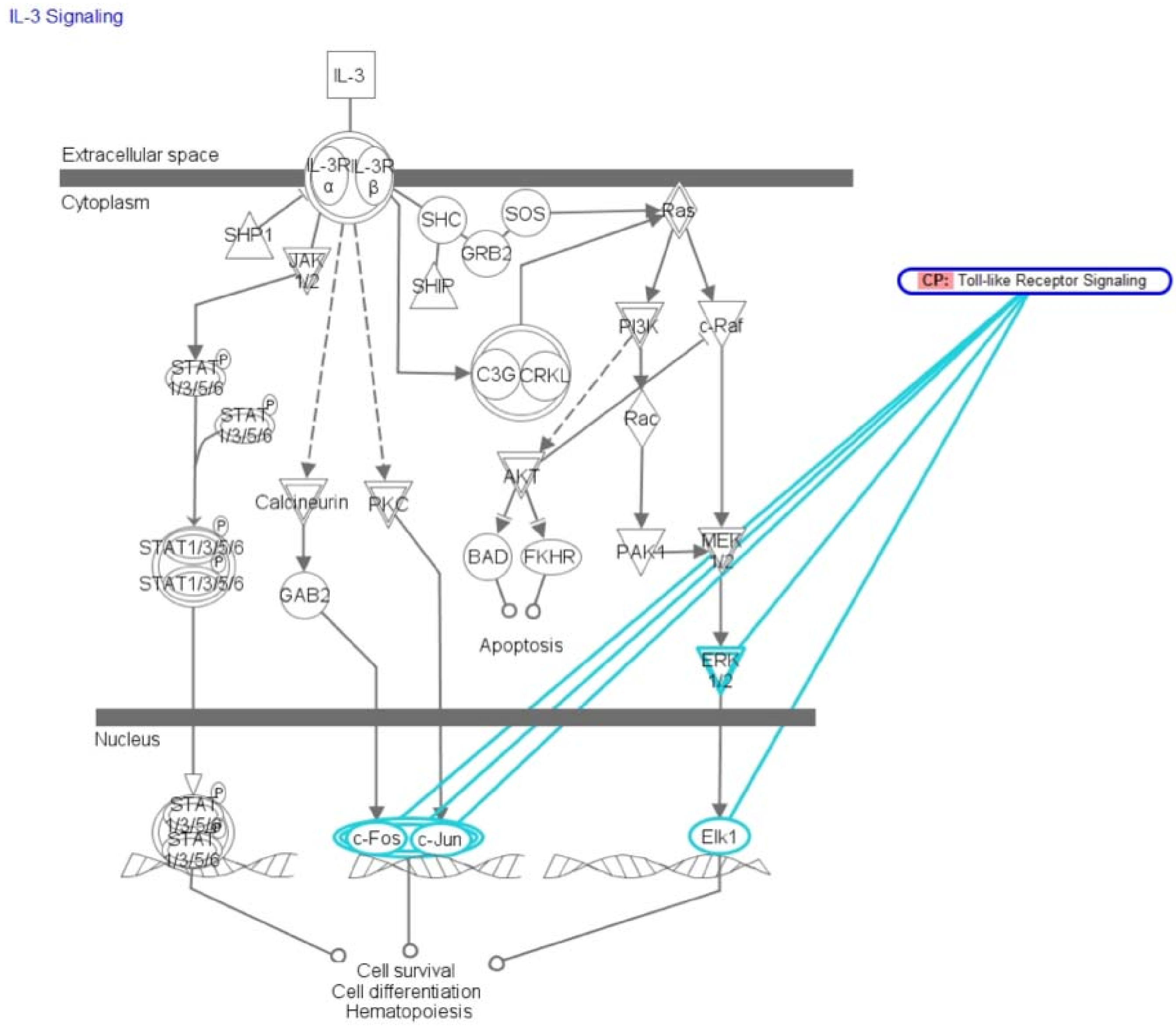

Supplementary Figure 5: The IL3 canonical pathway is represented with Ingenuity Pathway Analysis (IPA) software. The molecules inked with cyan lines to the box representing the Toll-like Receptor Signaling pathway are common to both pathways and are potentially levels at which signal interaction between IL3 and TLR ligands can occur.

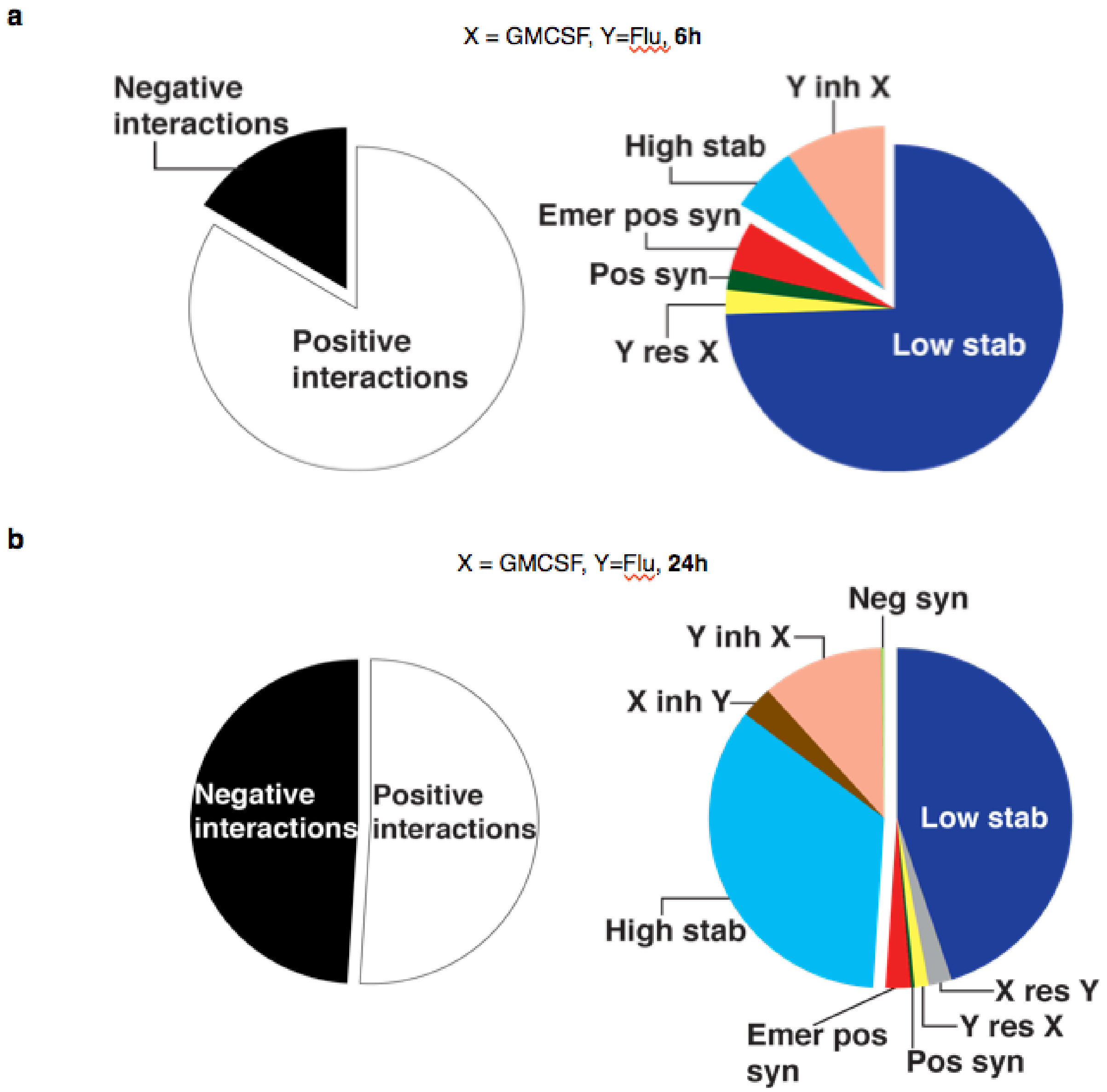

Supplementary Figure 6: Integration of GMCSF and Flu in human pDC. a, Distribution of Interaction modes a 6h **b**, Distribution of Interaction modes a 24h. Abbreviations used: low stab = low stabilization; X rest Y = GM-CSF restores Flu; Y rest X = Flu restores GM-CSF; pos syn = positive synergy; emer pos syn = emergent positive synergy; high stab = high stablization; X inh Y = GM-CSF inhibits Flu; Y inh X = Flu inh GM-CSF; neg syn = negative synergy.

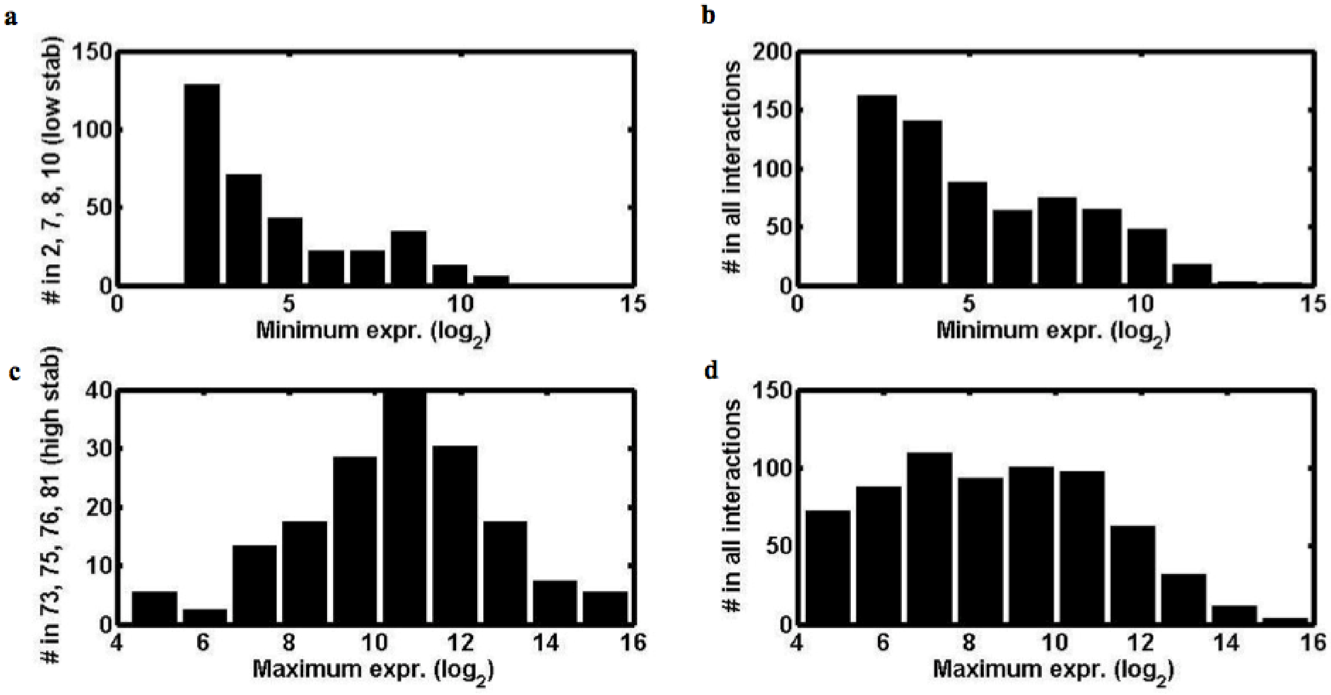

Supplementary Figure 7: A fraction of genes populating the modes Low and High Stabilization in the dataset X = IL3, Y = Flu at 6h does not seem to depend on technical limitations of the scanner. a, The frequency distribution of the minimum expression intensities within the modes Low Stabilization and **b**, the background provided by the minimum expression in all interaction profiles. **c**, The frequency distribution of the maximum expression intensities within the modes High Stabilization and **d**, the background provided by the maximum expression in all interaction profiles.

a

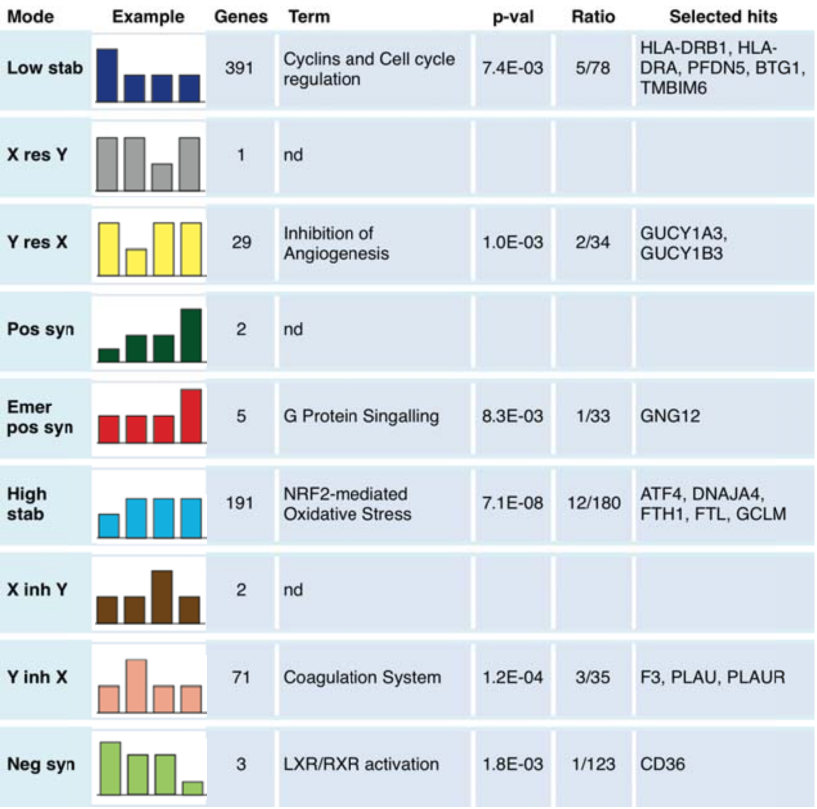

Mode	Example	Genes	Term	p-val	Ratio	Selected hits
Low stab		391	Cyclins and Cell cycle regulation	7.4E-03	5/78	HLA-DRB1, HLA-DRA, PFDN5, BTG1, TMBIM6
X res Y		1	nd			
Y res X		29	Inhibition of Angiogenesis	1.0E-03	2/34	GUCY1A3, GUCY1B3
Pos syn		2	nd			
Emer pos syn		5	G Protein Singalling	8.3E-03	1/33	GNG12
High stab		191	NRF2-mediated Oxidative Stress	7.1E-08	12/180	ATF4, DNAJA4, FTH1, FTL, GCLM
X inh Y		2	nd			
Y inh X		71	Coagulation System	1.2E-04	3/35	F3, PLAU, PLAUR
Neg syn		3	LXR/RXR activation	1.8E-03	1/123	CD36

b

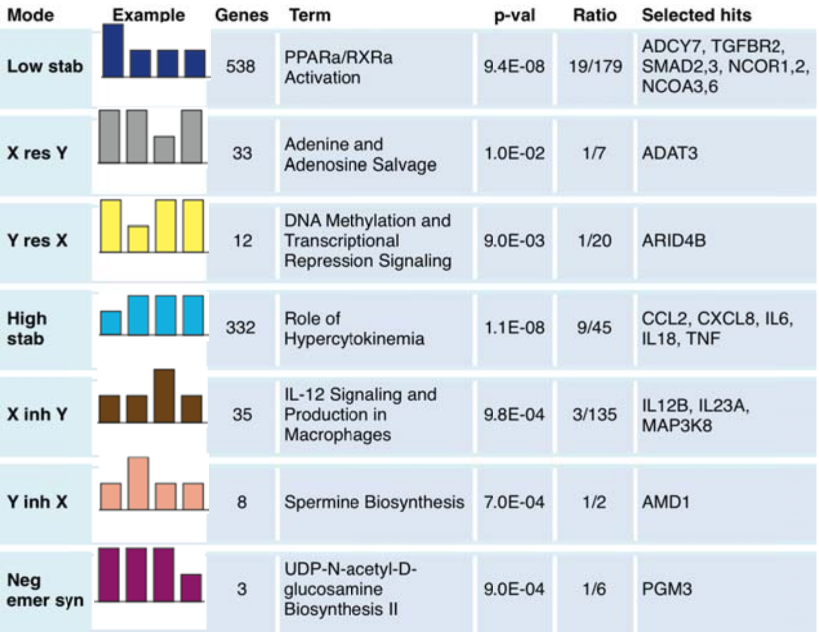

Mode	Example	Genes	Term	p-val	Ratio	Selected hits
Low stab		538	PPARa/RXRa Activation	9.4E-08	19/179	ADCY7, TGFBR2, SMAD2,3, NCOR1,2, NCOA3,6
X res Y		33	Adenine and Adenosine Salvage	1.0E-02	1/7	ADAT3
Y res X		12	DNA Methylation and Transcriptional Repression Signaling	9.0E-03	1/20	ARID4B
High stab		332	Role of Hypercytokinemia	1.1E-08	9/45	CCL2, CXCL8, IL6, IL18, TNF
X inh Y		35	IL-12 Signaling and Production in Macrophages	9.8E-04	3/135	IL12B, IL23A, MAP3K8
Y inh X		8	Spermine Biosynthesis	7.0E-04	1/2	AMD1
Neg emer syn		3	UDP-N-acetyl-D-glucosamine Biosynthesis II	9.0E-04	1/6	PGM3

Supplementary Figure 8: Top enriched canonical pathways or biological functions corresponding to each interaction mode from the datasets a, pDC integrating X=IL-3 and Y=Flu at 6h and **b**, Monocytes integrating X=LPS and Y=anti-TREM1 at 2h (Retrieved from GEO: GDS3499).
